# Inhibition of Pancreatic α-amylase by Resveratrol Derivatives: Biological Activity and Molecular Modelling Evidence for Cooperativity between Viniferin Enantiomers

**DOI:** 10.3390/molecules24183225

**Published:** 2019-09-05

**Authors:** Luce M. Mattio, Mauro Marengo, Chiara Parravicini, Ivano Eberini, Sabrina Dallavalle, Francesco Bonomi, Stefania Iametti, Andrea Pinto

**Affiliations:** 1 Department of Food, Environmental and Nutritional Sciences (DeFENS), University of Milan, Via Celoria 2, 20133 Milano, Italy; 2 Department of Pharmacological and Biomolecular Sciences (DiSFeB) & cDSRC, University of Milan, Via Balzaretti 9, 20133 Milano, Italy

**Keywords:** resveratrol, stilbenoids, pancreatic alpha-amylase, enzyme inhibition, molecular docking, food bioactives synergism

## Abstract

To improve the current understanding of the role of stilbenoids in the management of diabetes, the inhibition of the pancreatic α-amylase by resveratrol derivatives was investigated. To approach in a systematic way, the mechanistic and structural aspects of the interaction, potential bioactive agents were prepared as single molecules, that were used for the biological evaluation of the determinants of inhibitory binding. Some dimeric stilbenoids—in particular, viniferin isomers— were found to be better than the reference drug acarbose in inhibiting the pancreatic α-amylase. Racemic mixtures of viniferins were more effective inhibitors than the respective isolated pure enantiomers at an equivalent total concentration, and displayed cooperative effects not observed with the individual enantiomers. The molecular docking analysis provided a thermodynamics-based rationale for the measured inhibitory ability and for the observed synergistic effects. Indeed, the binding of additional ligands on the surface of the alpha-amylase was found to decrease the dissociation constant of inhibitors bound to the active site of the enzyme, thus providing a mechanistic rationale for the observed inhibitory synergies.

## 1. Introduction

Diabetes (type 1 or 2) is nowadays the most common serious metabolic disorder and one of the most challenging health problems [1,2]. Diabetes is characterized by persistent hyperglycaemia related to alterations in the glucose and lipid metabolism, including effects on key regulatory enzymes, and to associated oxidative stress. The management of blood glucose level may be accomplished by the use of oral hypoglycaemic drugs, such as biguanides, insulin secretagogues, and inhibitors of enzymes involved in the release of simple sugars from polysaccharides and in their uptake [3]. Pancreatic α-amylase is an endo-hydrolase acting on 1,4-glucosidic linkages in linear regions of suitable length in starch, glycogen, and in various oligosaccharides. The activity of the enzyme releases simpler sugars that are then converted into glucose for intestinal absorption by other enzymes, including the brush-border α-glucosidase. Therefore, the inhibition of α-amylase activity results in decreased bioavailability of oligosaccharides and absorbable sugars and, consequently, in a decrease of the postprandial hyperglycaemia. Alpha-amylase inhibitors (e.g., acarbose), competitively and reversibly inhibit the enzyme, but are reportedly associated with gastrointestinal side effects such as flatulence, abdominal pain, and diarrhoea [4,5].

Naturally occurring bioactives are currently considered precious elements of inspiration and an important source of structures for the development of novel nutraceuticals and pharmaceuticals [6]. The antioxidant activity and the related health benefits of dietary plant polyphenols are well documented, but there is an increasing evidence that polyphenols may have other beneficial effects in the management of diabetes, independent of their antioxidant capacities. In particular, they have been involved in direct modulation of the activity of key enzymes involved in the carbohydrate and lipid metabolism [7,8].

In this broad context, one particular group of the wood-derived natural products—namely, the stilbenoids family—has become increasingly popular, due to the wide spectrum of biological activities and to their intriguing molecular structures. Hydroxylated stilbenes—oligomers of resveratrol (3,5,4′-trihydroxystilbene) ranging from dimers to octamers—form one of the most interesting and therapeutically relevant groups of plant-derived polyphenols. Due to the wide variety of biological activities (anti-bacterial, anti-HIV, anti-inflammatory, and anti-proliferative) shown by stilbenoids, resveratrol-based medicinal chemistry has become rapidly evolving in the past decade, covering a broad range of therapeutic applications [9].

Numerous studies have demonstrated that resveratrol can have antidiabetic effects through diverse mechanisms that may address multiple molecular targets, resulting in a significant therapeutic action in the whole organism [10]. The complex physiological action of resveratrol as an antidiabetic agent has been attributed to its capacity to modulate different pathways and to its action on a diversity of molecular targets. A far-from-exhaustive list of protein targets for resveratrol includes phosphodiesterases, adenylyl cyclases, kinases, sirtuins, transcription factors, cytokines, etc. [11].

The vast range of activities and differing potencies attributed to resveratrol may be more easily accounted for by assuming that some of its possible oxidation products, including some of the naturally-occurring dimeric stilbenoids, were involved in eliciting specific effects. Indeed, the modes of action and the activities of resveratrol “in vivo” may be partially, or even solely, due to the activities of the oligomeric derivatives generated from the monomeric precursor in a biological environment. The products of stilbenoid oligomerization have a high structural complexity and a three-dimensional architecture that is not present in the resveratrol itself, thus leading to a higher probability for selective binding to a specific macromolecular target (be that of catalytic or regulatory proteins, receptors, or transporters).

To tackle in the most proper way the mechanistic and structural issues related to the specificity of these interactions, each potential bioactive agent needs to be prepared as a single molecule, and the individual species should be submitted to a biological evaluation separately. This “single molecule” approach used in this study has multiple advantages: (1) It allows to pinpoint the molecular determinants of the activity of individual chemical species; (2) it prevents the effects of possible competition among species present in variable concentration in the “natural” extracts most often used in this type of studies; (3) it makes it possible to highlight possible synergies among the individual bioactive species under investigation, as well as of therapeutically relevant synergies with synthetic compounds of pharmacological relevance.

In this report, in order to improve the current understanding of the determinants of the interaction of stilbenoids with one of key proteins in the glucose metabolism, we investigated the capacity of individual resveratrol derivatives to inhibit the pancreatic alpha-amylase, with the aim of providing structural insights into molecular features relevant to the biological activity of each chemical species. The issue of possible competition or synergy among the most active compounds was also tested by using appropriate combinations of pure enantiomers. Computational approaches based on molecular docking were used to elucidate the mode of binding of each of the active compounds to the target protein and to evaluate the thermodynamic stability of their various binding modes.

## 2. Results

### 2.1. Preparation of Individual Resveratrol Derivatives

**Figure 1:**
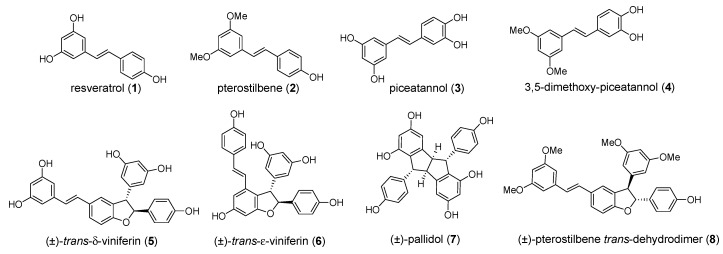
Structures of the representative stilbenoid monomers (**1**–**4**) and dimers (**5**–**8**).

To better understand the molecular determinants of the interaction of stilbenoids with the alpha-amylase and to clarify which physical properties can be exploited in the development of more potent congeners, we aimed to perform preliminary structure-activity relationship (SAR) studies. Our first objective was to have access to some representative monomers (compounds **1**–**4**, Figure 1).

**Figure 2:**
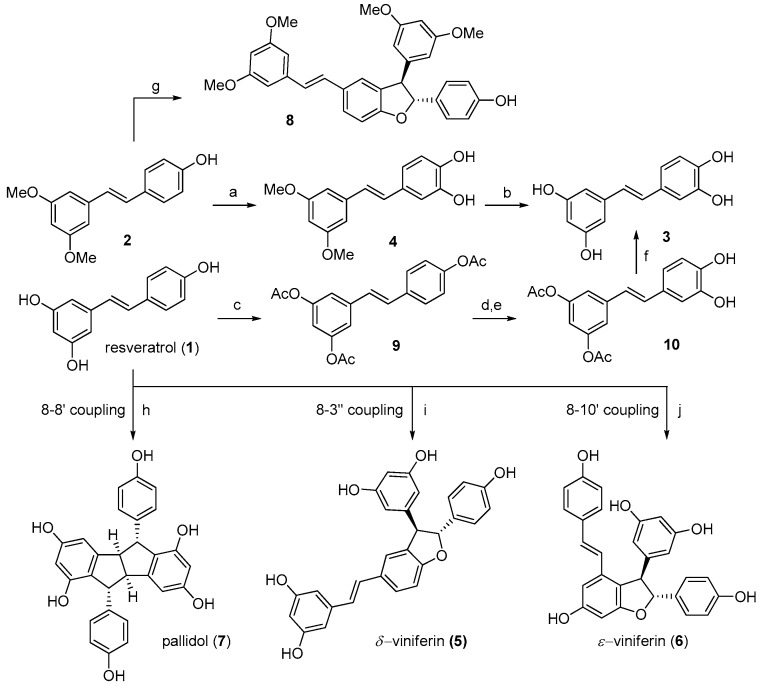
Synthesis of monomers **3**–**4** and dimers **5**–**8**. Reagents and conditions: a) 1-IBX, DMF, r.t., 80 min; 2-NaBH_4_ MeOH 0 °C, 62%; b) BBr_3_, DCM, −35 °C to r.t., N_2_, 22 h, 20%; c) Ac_2_O, TEA, DCM, r.t., overnight, 92%; d) CAL-B (*Candida antarctica* lipase), toluene/n-BuOH, 40 °C, 6 h, 81%; e) IBX, DMF, r.t., 80 min, 42%; f) NH_2_NH_2_, MeOH, r.t., 30 min, 81%; g) 1-HRP, 30 min, 40 °C, acetone: Citrate buffer pH 5 (1:1); 2-H_2_O_2_, 15 min, 40 °C, overall yield 61%; h) 1-HRP, 30 min, 40 °C, acetone: Phosphate buffer pH 8 (1:1); 2-H_2_O_2_, 90 min, 40 °C, overall yield 21%; i) 1 - HRP, 30 min, 40 °C, acetone: Citrate buffer pH 5 (1:1); 2-H_2_O_2_, 90 min, 40 °C, overall yield 49%; j) FeCl_3_ 6H_2_O, MeOH : H_2_O 1:1, 15%.

Resveratrol (**1**) and pterostilbene (**2**) are commercially available. Piceatannol (**3**) was obtained by the partial modification of a reported procedure [12]. Pterostilbene was oxidized with IBX in *N*, *N*-dimethylformamide (DMF), and the intermediate *O*-quinone was reduced with methanolic NaBH_4_ to obtain compound **4**, which was demethylated in a modest yield (20%) (Figure 2). Alternatively, compound **3** was obtained from peracetylated resveratrol **9**, which was selectively deacetylated by the *Candida antarctica* lipase to give compound **10**. The treatment of **10** with IBX and removal of the two acyl groups by the hydrazine monohydrate [13] finally gave piceatannol **3**.

Successively, we focused on dimeric compounds, whose three-dimensional architecture should improve the selectivity of their binding to an enzymatic target with respect to the planar monomers. However, low natural abundance and difficult extraction procedures prompted us to develop a synthetic strategy for dimers as well, based on a biomimetic approach. Resveratrol dimers are naturally obtained by an oxidative radical coupling. The regioisomeric dimerization modes constitute the foundation for the biosynthesis of a diverse collection of high-order resveratrol oligomers. Thus, a biomimetic synthetic strategy was followed [14], which allowed to obtain dimers (**5**–**8**, see Figure 1) in quantities suitable for their systematic biological evaluation.

*Trans*-δ-viniferin (**5**), pallidol (**7**), and pterostilbene-*trans*-dihydrodimer (**8**) were obtained from resveratrol (**1**) and pterostilbene (**2**) by treatment with horseradish peroxidase (HRP) and H_2_O_2_ [15,16] (Figure 2). *Trans*-ε-viniferin (**6**) was obtained through a metal-catalyzed oxidative coupling by subjecting resveratrol to one-electron oxidation by ferric ions (FeCl_3_·6H_2_O) [17].

**Figure 3:**
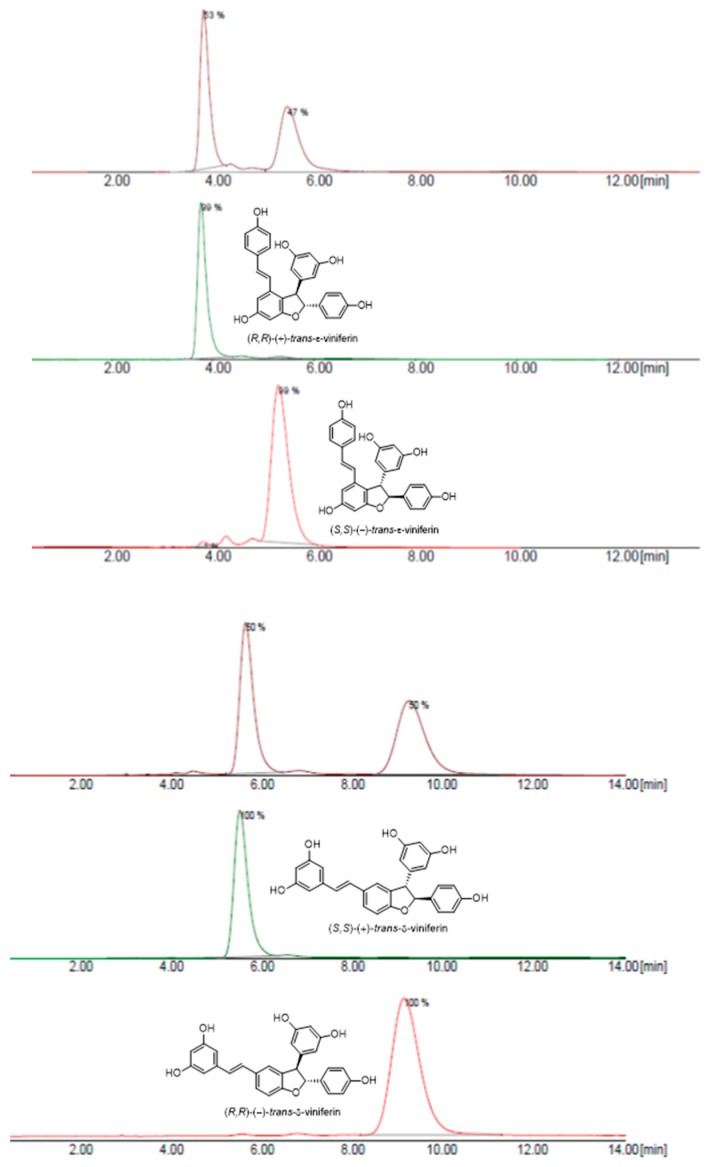
Chromatogram of the separation of two enantiomers of (±)-*trans*-δ-viniferin ((**5**), top) and (±)-*trans*-ε-viniferin ((**6**), bottom).

Regardless of whether the resveratrol oxidation was HRP-or metal-catalyzed [14], the first key step in dimerization is the formation of a phenoxy radical, which delocalizes due to the high conjugation of the stilbenoid nucleus and may be stabilized in position 8 or in position 3 and 10. Subsequent radical coupling (8-8’ coupling; 8-10’coupling; 8-3’ coupling) leads to the formation of very reactive *p*-quinone intermediates, which readily undergo intramolecular Friedel-Craft cyclization, giving respectively pallidol (**7**), *trans*-ε-viniferin (**6**) and *trans*-δ-viniferin (**5**). As a final step, the enantiomers of both the *trans*-δ-viniferin (**5**) and *trans*-ε-viniferin (**6**) were separated by the preparative chiral HPLC (Figure 3).

### 2.2. Alpha-amylase Inhibitory Activity

The monomeric and dimeric stilbenoids described above were tested for their ability to inhibit the enzyme α-amylase, using acarbose as a reference compound. The method used to evaluate the enzyme activity relies on a two-step procedure. In a first step, α-amylase acts on a modified p-nitrophenyl-α-D-maltoheptaoside, releasing products that may be hydrolyzed by an excess α-glucosidase present in the assay mixture, therefore allowing spectrophotometric quantitation of the released p-nitrophenolate anion. The data obtained with various inhibitors are expressed in comparison with control runs (all including 15% MeOH, as MeOH was the solvent used for preparing stock solutions of compounds **1**–**8**). None of the compounds tested here—at the concentrations used in this study—showed a significant inhibitory activity towards the purified α-glucosidase.

**Figure 4:**
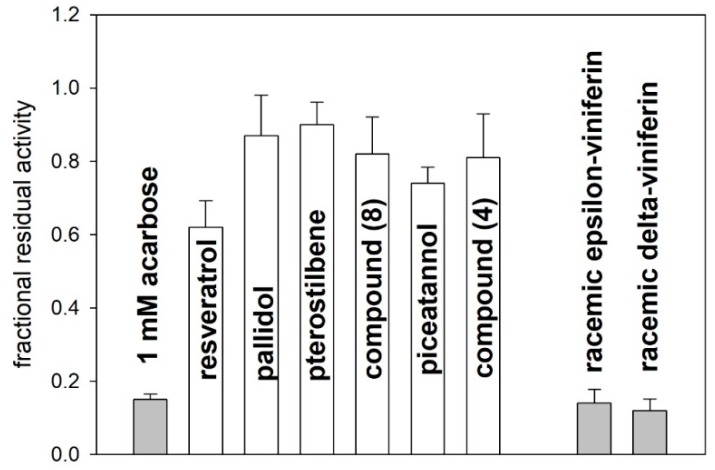
Inhibition of the pancreatic α-amylase by the various compounds presented in Figure 1, each at a 10 mM final concentration. The effect of a 1 mM acarbose is shown for comparison.

The results of a preliminary screening are reported in Figure 4. Among the tested compounds (all at a 10 mM final concentration), the four monomers (**1**–**4**) and pallidol (**7**) showed a modest inhibitory activity (21–45%) compared to acarbose (86% inhibition, at a 1 mM final concentration). Conversely, the inhibitory capacity of the dimeric species *trans*-δ-viniferin (**5**) and *trans*-ε-viniferin (**6**) was comparable (~90% inhibition at 10 mM) to that observed with a 1 mM acarbose. It is interesting to note that pterostilbene (**2**) and its dimer (**8**) decreased the enzyme activity of only 21% and 28%, respectively, indicating that the free hydroxyl groups in other similar species play a relevant role in the interaction with the enzyme.

Overall, the results in Figure 4 suggest that the racemic *trans*-δ-viniferin (**5**) and *trans*-ε-viniferin (**6**) were the most efficacious inhibitors of the pancreatic alpha-amylase. This observation prompted us to evaluate whether the inhibitory capacity of compounds (**5**) and (**6**) was correlated with their geometric features.

**Figure 5:**
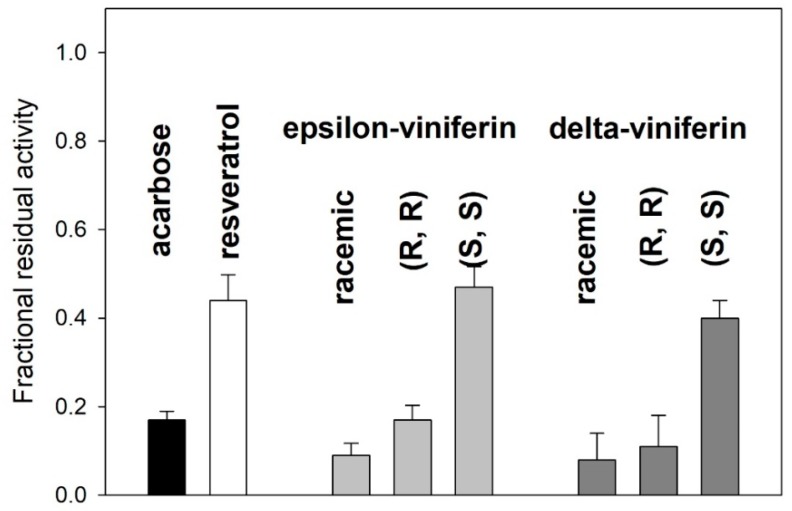
Residual enzyme activity in the presence of viniferin isomers, of their purified enantiomeric forms, and of their racemic forms. Final concentration of each inhibitor species (including acarbose and resveratrol) was 1 mM.

The racemic forms and the pure enantiomers of each compound were tested as for their inhibitory capacity at a 1 mM final concentration, always in comparison with a 1 mM acarbose. The data in Figure 5 show that the enantiomers with the (*R*, *R*) absolute configuration have a greater inhibitory efficacy than those of the (*S*, *S*) series, confirming that the inhibitory activity could be linked to the spatial arrangement of substituents on the 2,3-dihydrobenzofuran ring.

**Figure 6:**
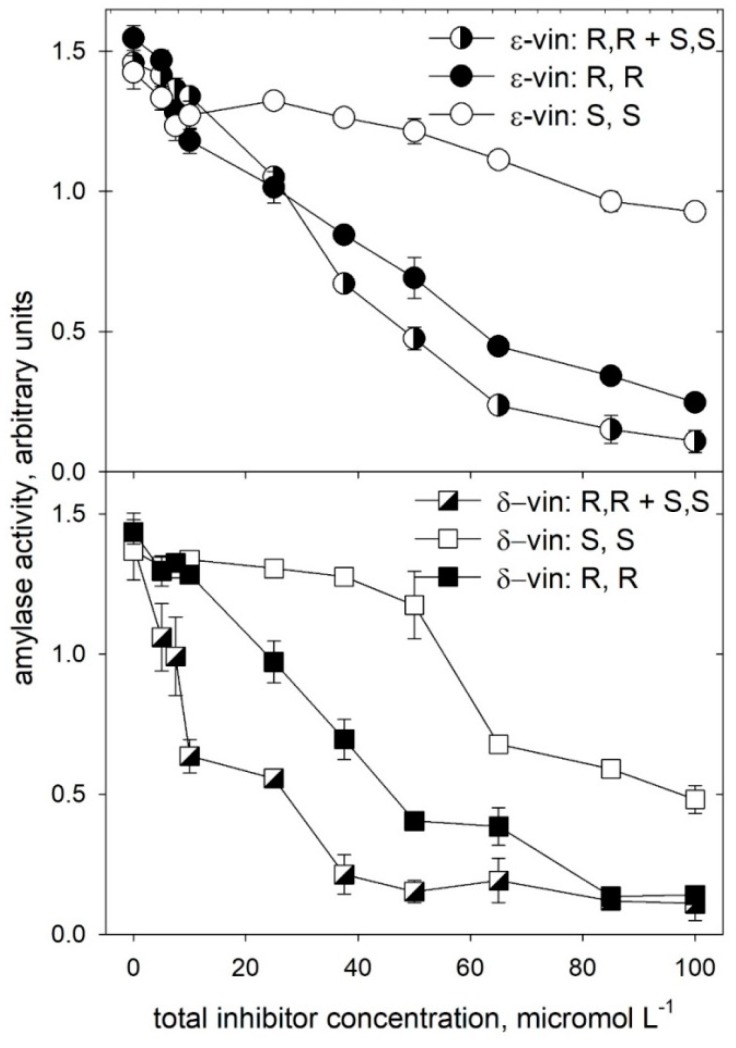
Concentration dependence of the α-amylase inhibition by purified enantiomeric forms of *trans*-δ-viniferin (**5**) and *trans*-ε-viniferin (**6**), and by equimolar mixtures of their purified enantiomers, simulating the presence of a racemic form of each species.

Moreover, quite surprisingly, the data in Figure 5 also show that the racemic forms of both the dimeric species have a greater inhibitory efficacy than their respective pure enantiomers, at equivalent total concentrations. To further elucidate this finding, inhibition studies were carried out over a range of inhibitor concentrations (zero to 0.1 mM, Figure 6) much lower than that used in previous experiments. These experiments were carried out using either purified enantiomers or their equimolar mixtures, meant to simulate the simultaneous presence of the racemic form of each viniferin while avoiding possible effects of minor impurities that could have been present in the original racemic mixtures.

The comparative analysis presented in Figure 6 confirm the higher inhibitory efficacy of *trans*-δ-viniferin (**5**) with respect to *trans*-ε-viniferin (**6**), as well as the different ability of the enantiomers with respect to inhibitory binding to the enzyme. In both cases, the “simulated” racemic forms of either molecule have a greater efficacy in inhibiting the enzyme than their respective pure enantiomers, at an equivalent total concentration.

**Figure 7:**
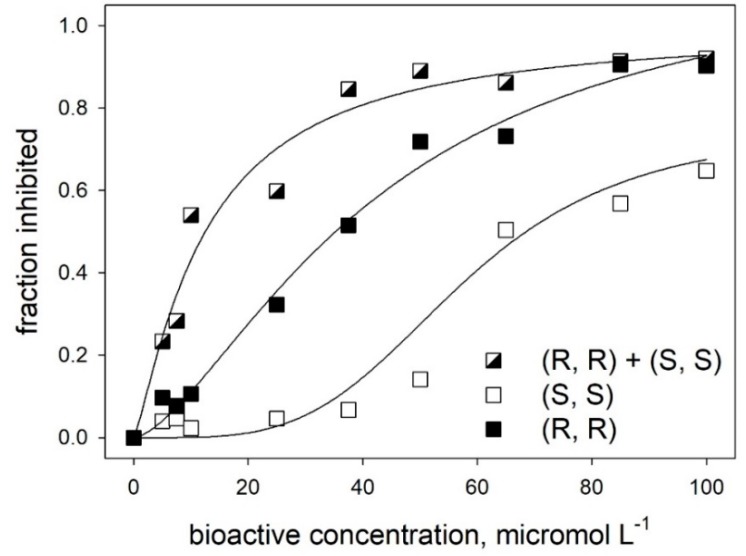
Hill plot for the inhibition of α-amylase by different enantiomeric forms of *trans-*δ-viniferin (**5**). Lines are the best fit to the experimental data, drawn by using a three-parameters Hill equation (y=a*x^n^H^/([K_i_]^n^H^ + x^n^H^).

The increased efficacy of the enantiomeric mixture is particularly evident in the case of *trans*-δ-viniferin (**5**), where the mode of binding of the two enantiomers may involve multiple and non-equivalent sites on the target protein. Indeed, as shown in Figure 7, the analysis of inhibitory binding of *trans*-δ-viniferin (**5**) through the Hill equation indicated facilitated binding of the (*S*, *S*) enantiomer at a high concentration. The markedly sigmoidal binding observed for the (*S*, *S*) enantiomer was characterized by a Hill cooperativity coefficient (n^H^) close to four and a relatively high average value of K_i_^app^ (58 μM). In contrast, values of n^H^ were close to unity for the *(R, R)* enantiomer (n^H^ = 1.5; K_i_^app^ = 43 μM) and for the high-affinity binding observed for an equimolar mixture of the (*R*, *R*) and (*S*, *S*) enantiomers (n^H^ = 1.2; K_i_^app^ = 12 μM).

Aside from confirming the presence of multiple binding sites for the enantiomeric forms of *trans*-δ-viniferin (**5**), with a distinct affinity and efficacy, the cooperative effects discussed above point to the facilitated binding when other binding sites on the target protein (not necessarily close to regions related to inhibitory effects) are occupied. This offers a possible explanation of the high inhibitory efficacy of enantiomeric mixtures (or of racemic forms) with respect to pure enantiomers. We hypothesize that sequential binding of a “low affinity” enantiomer to the enzyme having the “high affinity” enantiomer on the active site may result in a strong decrease in the dissociation constant of the high-affinity enantiomer. In other words, our hypothesis implies that the simultaneous binding of different enantiomers “locks” one of them into the alpha-amylase active site, decreasing its apparent dissociation constant K_i_^app^ and consequently increasing its inhibitory ability.

### 2.3. Molecular Docking Studies

**Figure 8:**
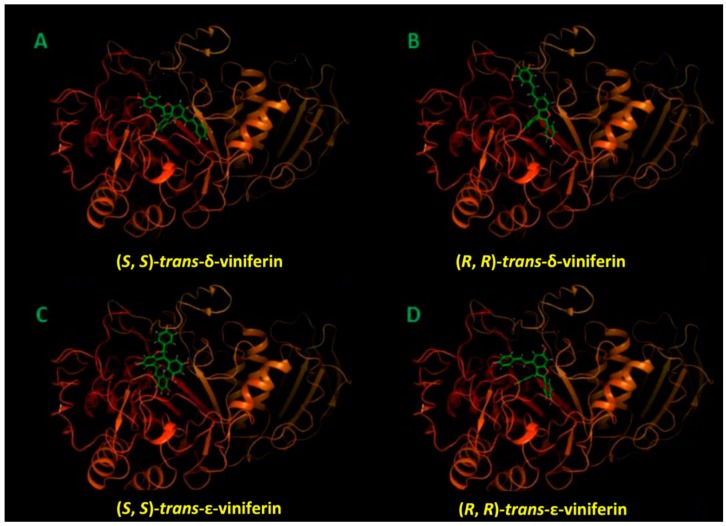
Top-scoring binding poses between the pig pancreatic α-amylase and the enantiomeric forms of the *trans*-viniferin species in the protein active site: A, (*S*, *S*)-*trans*-δ-viniferin; B, (*R*, *R*)-*trans*-δ-viniferin; C, (*S*, *S*)-*trans*-ε-viniferin; D, (*R*, *R*)-*trans*-ε-viniferin.

**Figure 9:**
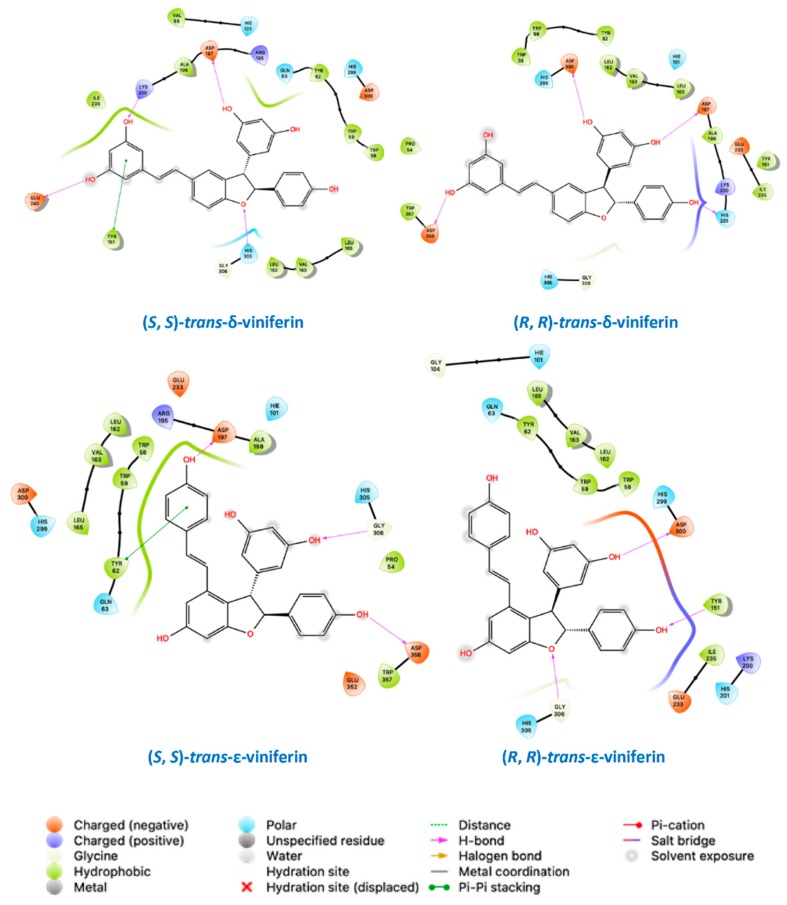
Ligand interaction diagram for the molecular docking top-scoring complexes between the pig pancreatic α-amylase and various enantiomeric forms of the *trans*-viniferin isomers in the protein active site. Interactions are recapitulated in the figure legend.

Supporting evidence for the data interpretation offered above came through molecular docking studies. The various panels of Figure 8 show the top-scoring binding pose for individual enantiomeric species of compounds (**5**) and (**6**) in the amylase active site, whereas their detailed molecular recognition mechanism is reported in the schematic views presented in Figure 9. In short, binding free energy values obtained from molecular docking confirm that the amylase active site may accommodate all these species, although with different affinities, as reported in Supporting Information Table S1. The affinities of the investigated compounds for the enzyme, as assessed through the docking scores, are consistent with the efficacy of individual species as inhibitors, as made evident by the enzymatic assays reported and commented in the previous subsection.

Docking studies (Figure 8; Figure 9, and Table S1 in the Supporting Information) highlight the relevance of hydroxyl groups in the interaction with the enzyme, explaining the virtual absence of inhibitory effects for some of the derivatives under scrutiny, even at concentration orders of magnitude higher than those where viniferins proved effective.

In this regard, not all phenols were created equal, since each of the species with a high inhibitory capacity has a different set of protein residues as specific interactors, as made evident by the schematics presented in Figure 9. Quite expectedly, the set of protein interactors and the nature of the interactions was different for the two viniferin isomers, in consideration of their vastly different overall geometry. Perhaps more excitingly, enantiomers of each species were found to differ in their mode of docking to the protein. The presence of differences in the spatial arrangement of substituents at their stereogenic centers resulted in a completely different set of protein interactors for other-structurally similar-portions of the molecule.

In more general terms, Figure 8 and Figure 9 suggest that each of the most efficient inhibitors has a specific binding mode. Taking into account the specificity of the binding (as indicated by computational data) and the efficacy of individual stilbenoids derivatives (see Figure 1), it seems that the most active species described in this study (with K_i_^app^ in the 10–20 micromolar range) do not just behave as “hydrophobic plugs” with respect to limiting access of the substrate to the active site of the enzyme.

On the other hand, computational data point out the ability of the α-amylase active site to accommodate compounds with sensibly different geometric features. Active site binding in the arrangements proposed here takes advantage of a pocket in the protein interior—in close proximity of the active site, which is supposedly close to the surface of the protein and meant to harbour large and hydrophilic species—able to accommodate with relative ease the bulky substituents on the 2,3-dihydrobenzofuran core of stilbenoid dimers.

Molecular modelling was also applied to explore the possibility of gaining mechanistic insights into the “racemic effect” amply discussed in a previous subsection when commenting inhibition data, and took into account the minimum energy mode of simultaneous binding of different enantiomers of *trans*-δ-viniferin (**5**) to α-amylase. Our modelling approach started from the top-scoring complexes of the *trans*-δ-viniferin enantiomers presented in Figure 8 and Figure 9, in order to explain the synergistic inhibition effect of this racemic mixture on the pancreatic α-amylase.

**Figure 10:**
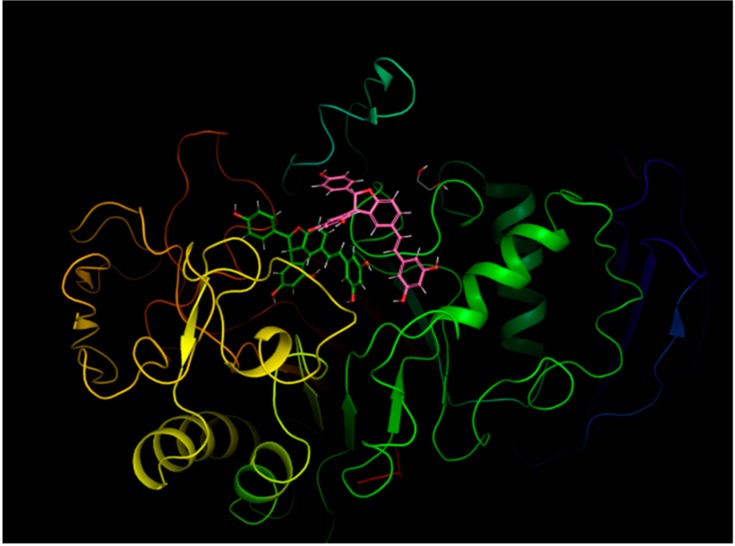
Top scoring pose of (*R*, *R*)-*trans*-δ-viniferin to the pig pancreatic alpha amylase with (*S*, *S*)-*trans*-δ-viniferin already bound to its active site.

In the proposed model (Figure 10), (*S*, *S*)-*trans*-δ-viniferin is the most affine compound for the enzyme active site. The possibility for its enantiomeric form, (*R*, *R*)-*trans*-δ-viniferin, to bind in a more external protein site and to stabilize the enzyme complex with (*S*, *S*)-*trans*-δ-viniferin has been demonstrated to be thermodynamically feasible. As a result, simultaneous binding of different enantiomeric forms stabilizes the resulting complex, and thus promotes an overall increase in the inhibitory capacity.

Molecular docking calculations, followed by MM, showed that the involved change in the binding free energy computed through the MM-GBSA approach (namely, −66.54 kcal/mol for (*S*, *S*)-*trans*-δ-viniferin in the binding site with externally bound (*R*, *R*)-*trans*-δ-viniferin versus −44.58 kcal/mol for (*S*, *S*)-*trans*-δ-viniferin in the active site by itself; corresponding to a ΔΔG of 21.96 kcal/mol) were fully compatible with the changes in the inhibitory capacity observed in the experiments reported in the various panels of Figure 6 and analyzed in terms of coperativity through the Hill plot presented in Figure 7.

## 3. Discussion

The overall evidence gathered in this study highlights that: 1) Some dimeric stilbenoids species—most notably, the naturally occurring isomers of *trans*-viniferins—may be way more efficacious than acarbose in inhibiting the pancreatic α-amylase; 2) the geometric features of individual viniferin isomers affect their inhibitory ability; 3) racemic mixtures of either the *trans*-δ- or *trans*-ε-viniferin are more effective inhibitors than the respective isolated pure enantiomers; 4) the molecular docking analysis provides thermodynamics-based evidence for the observed inhibitory ability (or lack of it) of the various stilbenoids considered in this study, as well as a mechanistic rationale for the synergistic effect observed in racemic mixtures of the *trans*-viniferin isomers.

Point 1 and 2 in the above list are of particular interest, also because intestinal concentrations of the most efficacious stilbenoid dimers may be higher than expected for a given food on a composition-only basis, due to their poor absorption at the mucosal level. In this frame, it is worth also reminding that the dimeric stilbenoid species may form through a simple metabolic transformation of their precursors, that are usually much more abundant than oligomers in common foods and beverages. Notably, viniferins have been reported to represent up to 20% of the total stilbenoids in wine [18]. In addition, procedures are available for the selective recovery of viniferins from food-processing byproducts [19]. However, viniferins recovered from byproducts are a mixture of isomers, with a relative abundance of the individual species being dependent on the source.

Point 3 in the list above represents the most novel—and most unexpected—outcome of our studies. As a matter of fact, synergistic effects appear to be at work in many of the reported protein-phenolics inhibitory interactions [20,21]. However, neither the nature of the involved chemical species nor the molecular basis of these effect appears to have been investigated in sufficient detail, in particular when chemically similar components could be involved. To our best knowledge, this is the first report in which chemically pure species were used to carry out detailed analysis of the geometric and structural determinants of these synergistic effects. From a practical standpoint, the observation that the racemic mixtures of dimeric stilbenoids are more effective than the isolated enantiomers offers circumstantial support for the “food better than pills” intervention strategies, and could offer useful guidelines for the structural design of molecules with improved efficacy as well as for their usage and dosage, either alone or in combination.

In this frame, it is worth noting that preliminary experiments indicate that dimeric stilbenoids do not compete with acarbose in the inhibition of the pancreatic α-amylase. On the contrary, the evidence gathered so far points to a possible synergy among various classes of the inhibitory species, even when apparently unlikely because of their remarkable structural differences. The possible occurrence of a similar synergism in other enzymes relevant to the human health seems a research territory still unchartered and much worth exploring.

Finally, point 4 in the list above underlines the relevance of molecular docking studies in this type of investigation, in particular when used in combination with the biological activity data. In the case reported here, this combination of approaches offers a clue for explaining the synergistic effects mentioned above, and could provide useful hints as for their preventive or therapeutic use.

## 4. Materials and Methods

### 4.1. Chemistry

Unless otherwise specified, chemicals were from Sigma-Aldrich (Milan, Italy), as were the *Candida antarctica* lipase and the horseradish peroxidase used for some of the synthetic approaches reported here. All reagents and solvents were of reagent grade or were purified by standard methods before use. Melting points were determined on a model B-540 Büchi apparatus and are uncorrected. Optical rotation determinations were carried out using a Jasco P-1010 spectropolarimeter (Jasco Europe, Cremella, Italy), coupled with a Haake N3-B thermostat. NMR data were acquired using a Varian Mercury-300 MHz spectrometer (Varian, Palo Alto, CA, USA). Chemical shifts (δ values) and coupling constants (*J* values) are given in ppm and Hz, respectively. Chiral HPLC analyses were performed using a Kromasil 5-AmyCoat column (4.6 mm i.d. × 250 mm, Nouryon Separation Products, Bohus, Sweden), fitted to a Jasco PU-980 pump and a Jasco UV-975 detector. Runs were carried out in 60/40 (*v/v*) hexane/iPrOH + 0.1% TFA at a flow rate of 1 mL min^-1^, monitoring the eluate at 280 nm. Preparative chiral HPLC was performed with a Kromasil 5-AmyCoat column (21.2 i.d. × 250 mm), fitted to a 1525 Extended Flow Binary HPLC pump and a Waters 2489 UV/Vis detector (both from Waters, Milan, Italy). The solvent was 60/40 (*v/v*) hexane/iPrOH + 0.1% TFA, at a flow rate of 15 mL min^-1^, monitoring the eluate at 280 nm. Isolation and purification of the compounds were performed by a flash column chromatography on silica gel 60 (230–400 mesh). The analytical thin-layer chromatography (TLC) was conducted on Supelco TLC plates (silica gel 60 F_254_, aluminum foil). Structural characterizations of the various stilbenoid derivatives mentioned above are detailed in the Supplementary materials and are in accordance with literature reports [22,23,24,25,26,27,28,29].

### 4.2. Enzymatic Inhibition 

Porcine pancreatic α-amylase [EC 3.2.1.1] was from Sigma-Aldrich (Milan, Italy). Activity was monitored by using the K-AMYLSD kit from Megazyme (Bray, Ireland), with modifications. An aliquot of α-amylase (0.03 mL, 0.1 μM in 50 mM phosphate buffer, pH 6.8) was added to 0.120 mL of 50 mM phosphate buffer, pH 6.8, in a microtiter plate well. The investigated compounds where then added as methanolic solutions (0.03 mL) of appropriate concentration. The reaction was started by adding 0.02 mL of the substrate solution provided with the kit, resulting in a final concentration of 0.4 μM substrate. The kit-provided substrate solution also contained an excess of thermostable bacterial α-glucosidase, that is required for the release of the *p*-nitrophenolate anion from the *p*-nitrophenyl glucosides produced by the activity of α-amylase on the chemically modified maltoheptaoside. At appropriate times, the reaction was stopped by adding 0.1 mL of 0.2 M Na_2_CO_3_, and the absorbance was read in a microplate reader set at 405 nm. Blanks were prepared in the absence of the pancreatic α-amylase. Controls were otherwise complete reaction mixtures, containing 15% methanol (*v/v*), but no bioactive species. Acarbose (from a stock solution in methanol) was used as the reference inhibitor. A minimum of three independent replications was carried out for each assay.

### 4.3. Computational Procedures

All the computational procedures were carried out with a Schrödinger Maestro BioLuminate v. 2019-01 (http://schrodinger.com) with the OPLS3e force field, as previously described [30]. The crystallographic structure of the pig pancreatic alpha amylase was downloaded from RCSB PDB (id: 1HX0) and was checked, fixed, and relaxed through the Protein Preparation Wizard. The *trans*-viniferin species and enantiomeric form database was sketched and prepared with the LigPrep, carefully checking the stereocenters. Molecular docking was carried out through the Glide Ligand Docking with a standard precision (SP) according to a well-established protocol [31]. The active site was identified through the Site Map tool and confirmed according to the annotations of the UniProt pig pancreatic alpha amylase entry (AMYP_PIG). The prime MM-GBSA was run in order to estimate the binding energy of the (*S*, *S*)-*trans*-δ-viniferin to the active site of the pig pancreatic alpha amylase in the presence and absence of (*R*, *R*)-*trans*-δ-viniferin.

### 4.4. Statistical Analysis

The analysis of variance on the enzymatic inhibition data was performed adopting the least significant difference (LSD). Data were processed by using the Statgraphics XV, version 15.1.02 (StatPoint, Warrenton, VA, USA). The appropriate non-linear regression routines in SigmaPlot (rev. 10, Jandel Scientific, San Rafael, CA, USA) were used for graphical and numerical analysis of the inhibition data.

## Supplementary Materials

The following are available online, Characterization of compounds **3**–**10**; Table S1: Docking score of top scoring complexes.
