# Supplementary material for: Inhibition of Pancreatic α-amylase by Resveratrol Derivatives: Biological Activity and Molecular Modelling Evidence for Cooperativity between Viniferin Enantiomers
Source: Molecules. 2019 Sep 5;24(18):3225. doi: 10.3390/molecules24183225 (PMC6766848; doi:10.3390/molecules24183225)
Supplement: Supplementary file 1 [file molecules-24-03225-s001.pdf]

Supporting Information

Inhibition of pancreatic α-amylase by resveratrol derivatives: biological activity and molecular modelling evidence for co-operativity between viniferin enantiomers

Luce M. Mattio, Mauro Marengo, Chiara Parravicini, Ivano Eberini, Sabrina Dallavalle, Francesco Bonomi, Stefania Iametti, Andrea Pinto

Corresponding author: E-mail: andrea.pinto@unimi.it

Table of contents

Characterization of compounds **3-10** page **S2**

Separation of enantiomers (2*S*,3*S*)-(+)-**5** and (2*R*,3*R*)-(–)-**5** page **S7**

Separation of enantiomers (2*R*,3*R*)-(+)-**6** and (2*S*,3*S*)-(–)-**6** page **S7**

References page **S8**

Table S1. Docking score of top scoring complexes page **S9**

Characterization of compounds 3–10

Chemicals

All reagents and solvents were purchased from Sigma-Aldrich. Flash column chromatography was performed on Merck SilicaGel (200–400 mesh). Compounds were synthesized according to synthetic procedures already reported in the literature [22–29]. ^{1}H NMR spectra were recorded with a Varian Mercury 300 (300 MHz) spectrometer and are in agreement with literature data [22–29]. Chemical shifts (δ) are expressed in ppm, and coupling constants (*J*) are expressed in Hz.

(*E*)-4-(3,5-dimethoxystyryl)benzene-1,2-diol (4) [22].

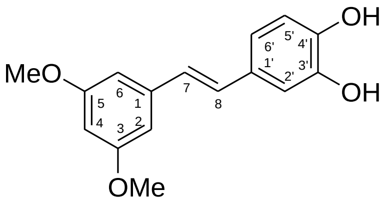

Pale yellow solid. M.p.: 112–115 ℃. Yield: 62%. R$_f$: 0.28 (Cyclohexane/EtOAc 7:3). ^{1}H NMR (300 MHz, methanol-*d$_4$*) δ 7.05 (d, *J* = 1.8 Hz, 1H, H-2'), 6.98 (d, *J* = 16.2 Hz, 1H, H-7), 6.86 (dd, *J* = 1.8, 8.1 Hz, 1H, H-6'), 6.83 (d, *J* = 16.2 Hz, 1H, H-8), 6.74 (d, *J* = 8.1 Hz, 1H, H-5'), 6.64 (d, *J* = 1.8 Hz, 2H, H-2 and H-6), 6.35 (t, *J* = 1.8 Hz, 1H, H-4), 3.80 (s, 6H, O-$\underline{CH_3}$) ^{13}C NMR (75 MHz, acetone-*d6*) δ 161.0, 143.9, 143.8, 139.7, 130.9, 128.9, 127.1, 120.42, 115.7, 113.2, 104.6, 99.9, 55.7.

(*E*)-5-(4-acetoxystyryl)-1,3-phenylene diacetate (**9**) [**23**].

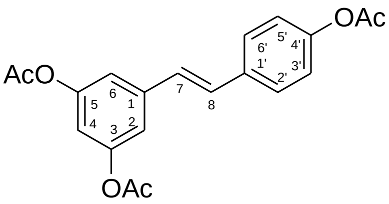

White solid. M.p.: 110-112 ℃. Yield: 92%. R$_f$: 0.40 (Cyclohexane/EtOAc 7:3). ^{1}H NMR (300 MHz, chloroform-*d*) δ 7.50–7.46 (m, 2H, H-2' and H-6'), 7.12 (d, *J* = 1.7 Hz, 2H, H-2 and H-6), 7.11–7.06 (m, 2H, H-3' and H-5'), 7.06 (d, *J* = 16.2 Hz, 1H, H-7), 6.96 (d, *J* = 16.2 Hz, 1H, H-8),

6.82 (t, *J* = 1.7 Hz, 1H, H-4), 2.31 (s, 9H, 3 x OCO<u>CH</u>$_3$). ^{13}C NMR: (75 MHz, DMSO-d_6) δ: 169.1, 168.9, 151.1, 150.2, 139.3, 134.2, 129.4, 127.6, 126.7, 122.1, 117.1, 114.9, 20.8, 20.7.

(*E*)-5-(3,4-dihydroxystyryl)-1,3-phenylene diacetate (**10**) [**24**].

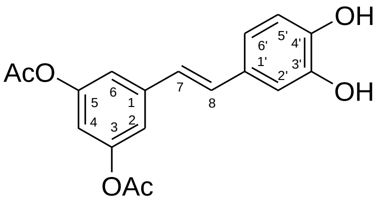

Yellow oil. Yield: 42%. R_f: 0.38 (DCM/EtOAc 95:5). ^{1}H NMR (300 MHz, acetone-*d6*) δ 8.01 (brs, 2H, 2 × OH), 7.19 (d, *J* = 1.7 Hz, 2H, H-2 and H-6), 7.16 (d, *J* = 16.2 Hz, 1H, H-7), 7.13 (d, *J* = 1.8 Hz, 1H, H-2'), 6.98 (d, *J* = 16.2 Hz, 1H, H-8), 6.97 (dd, *J* = 1.8, 8,1 Hz, 1H, H-6'), 6.83 (d, *J* = 8,1 Hz, 1H, H-5'), 6.81 (dt, *J* = 1.7 Hz, 1H, H-4), 2.28 (s, 6H, 2 × OCO<u>CH</u>$_3$). ^{13}C NMR: (75 MHz, acetone-*d6*) δ 170.0, 151.2, 144.2, 140.4, 130.3, 129.7, 123.9, 120.1, 116.9, 115.3, 113.6, 113.1, 21.1.

(*E*)-4-(3,5-dihydroxystyryl)benzene-1,2-diol (Piceatannol) (**3**) [**25**].

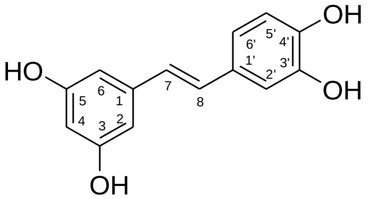

White solid. M.p.: 228-229 ℃. Yield: 81%. R_f: 0.31 (DCM/MeOH 9:1). ^{1}H NMR (300 MHz, methanol-*d4*) δ 7.97 (brs, 4H, 4 x OH), 6.98 (d, *J* = 1.8 Hz, 1H, H-2'), 6.88 (d, *J* = 16.5 Hz, 1H, H-7), 6.82 (dd, *J* = 8.1, 1.8 Hz, 1H, H-6'), 6.74 (d, *J* = 16.5 Hz, 1H, H-8), 6.73 (d, *J* = 8.1 Hz, 1H, H-5'), 6.44 (d, *J* = 1.8 Hz, 2H, H-2 and H-6), 6.16 (t, *J* = 1.8 Hz, 1H, H-4). ^{13}C NMR (75 MHz, acetone-d_6) δ 158.6, 145.3, 145.2, 140.2, 130.0, 128.8, 126.2, 119.5, 115.7, 113.2, 105.2, 102.1.

(*E*)-5-((±)-2-(3-(3,5-dihydroxyphenyl)-2-(4-hydroxyphenyl)-2,3-dihydrobenzofuran-5 yl)vinyl)benzene-1,3-diol (δ-viniferin) (±)-**5** [**26**].

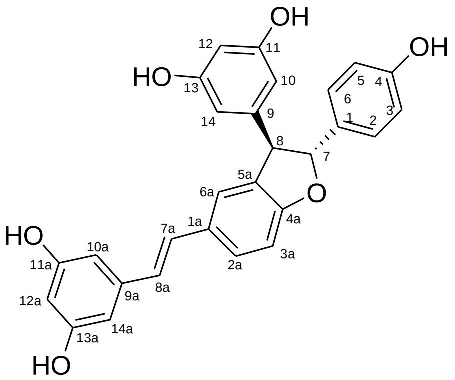

Yellow amorphous solid. Yield: 49%. R_f: 0.25 (DCM/MeOH 9:1). ^{1}H NMR (300 MHz, methanol-d_4) δ 7.36 (dd, *J* = 8.1, 1.8 Hz, 1H, H-2a), 7.20–7.14 (m, 2H, H-2 and H-6), 7.18 (d, *J* = 1.8 Hz, 1H, H-6a), 6.98 (d, *J* = 16.2 Hz, 1H, H-7a), 6.85 (d, *J* = 8.1 Hz, 1H, H-3a), 6.80–7.74 (m, 2H, H-3 and H-5), 6.78 (d, *J* = 16.2 Hz, 1H, H-8a), 6.43 (d, *J* = 2.2 Hz, 2H, H-10a and H-14a), 6.19 (t, *J* = 2.2 Hz, 1H, H-12), 6.14 (t, *J* = 2.2 Hz, 1H, H-12a), 6.12 (d, *J* = 2.2 Hz, 2H, H-10a and H-14a), 5.38 (d, *J* = 8.4 Hz, 1H, H-7), 4.40 (d, *J* = 8.4 Hz, 1H, H-8). ^{13}C NMR: (75 MHz, methanol-d_4) δ 161.0; 159.0; 159.7; 158.8; 145.4; 141.2; 132.9, 132.4; 132.4; 129.4; 128.7; 127.5; 124.2; 116.3; 110.4; 107.8; 105.8; 102.7; 102.5; 94.9; 58.0.

(*E*)-5-((±)-6-hydroxy-2-(4-hydroxyphenyl)-4-(4-hydroxystyryl)-2,3-dihydrobenzofuran-3-yl)benzene-1,3-diol (*trans*-ε-viniferin) (±)-**6** [**27**].

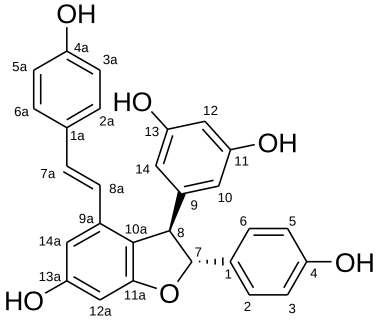

Light green amorphous solid. Yield: 15%. R_f: 0.30 (cyclohexane/acetone 3:2) - 0.42 (H_2O/MeOH 2:3). ^{1}H NMR (300 MHz, Methanol-d_4) δ 7.17–7.10 (m, 2H, H-2a and H-6a), 7.07–7.01 (m, 2H, H-2 and H-6), 6.82 (d, *J* = 16.2 Hz, 1H, H-8a), 6.78–6.73 (m, 2H, H-3a and H-5a), 6.67–6.62 (m, 2H, H-3 and H-5), 6.63 (d, *J* = 2.1 Hz, 1H, H-14a), 6.56 (d, *J* = 16.2 Hz, 1H, H-7a), 6.24 (d, *J* = 2.1 Hz, 1H, H-12a), 6.20–6.14 (m, 3H, H-12, H-10 and H-14), 5.36 (d, *J* = 6.6 Hz, 1H, H-7), 4.34 (d, *J* = 6.6 Hz, 1H, H-8). ^{13}C NMR (100 MHz, methanol-d_4) *δ*: 162.8, 160.1, 159.8, 159.7, 158.5,

158.4, 147.4, 137.0, 136.9, 133.9, 130.5, 130.4, 128.8, 128.2, 123.8, 123.7, 120.1, 116.4, 116.3, 107.5, 104.4, 102.2, 96.9, 94.9, 58.3.

(±)-5,10-bis(4-hydroxyphenyl)-4b,5,9b,10-tetrahydroindeno[2,1-a]indene-1,3,6,8-tetraol (pallidol) (±)-**7** [**28**].

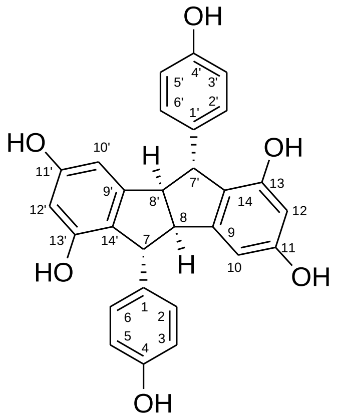

Brown solid. M.p.: 296 ℃ dec. Yield: 21%. R*f*: 0.15 (DCM/MeOH 9:1). ^{1}H NMR (300 MHz, methanol-*d4*) δ 6.99–6.87 (m, 4H, H-2, H-6, H-2', H-6'), 6.68–6.62 (m, 4H, , H-3, H-5, H-3', H-5'), 6.52 (d, *J* = 2.1 Hz, 2H, H-10 and H-10'), 6.10 (d, *J* = 2.1 Hz, 2H, H-12 and H-12'), 4.46 (s, 2H, H-7 and H-7'), 3.72 (s, 2H, H-8 and H-8'). ^{13}C NMR (75 MHz, acetone-*d6*) δ 159.3, 156.3, 155.3, 150.3, 137.7, 129.0, 123.2, 115.8, 103.3, 102.5, 60.5, 53.9.

(*E*)-4-((±)-3-(3,5-dimethoxyphenyl)-5-(3,5-dimethoxystyryl)-2,3-dihydrobenzofuran-2-yl)phenol (±)-**8** [**29**].

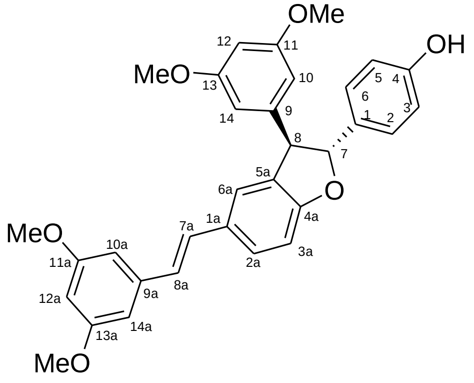

White amorphous solid. Yield: 61%. R*f*: 0.2 (cyclohexane/EtOAc 3:1). ^{1}H NMR (300 MHz, chloroform-*d*) δ 7.37 (dd, *J* = 8.3, 0.8 Hz, 1H, H-2a), 7.24–7.19 (m, 3H, H-2, H-6 and H-6a), 7.02

(d, *J* = 16.2 Hz, 1H, H-8a), 6.92 (d, *J* = 8.3 Hz, 1H, H-3a), 6.88–6.78 (m, 3H, H-3, H-5 and H-7a), 6.62 (d, *J* = 2.2 Hz, 2H, H-10a and H-14a), 6.41 (t, *J* = 2.2 Hz, 1H, H-12a), 6.36 (t, *J* = 2.2 Hz, 1H, H-12), 6.34 (d, *J* = 2.2 Hz, 2H, H-10 and H-14), 5.51 (d, *J* = 8.4 Hz, 1H, H-7), 4.48 (d, *J* = 8.4 Hz, 1H, H-8), 3.81 (s, 6H, 2 × O$\underline{CH_3}$), 3.75 (s, 6H, 2 × O$\underline{CH_3}$). ^{13}C NMR (125 MHz, chloroform-*d*) δ 161.2, 161.0, 159.8, 156.0, 144.0, 139.8, 132.6, 132.3, 132.1, 130.9, 130.8, 129.1, 128.8, 128.1, 127.6, 126.3, 123.2, 115.6, 109.8, 106.5, 104.3, 99.8, 57.9, 55.5.

5-((*E*)-2-((2*S*,3*S*)-3-(3,5-dihydroxyphenyl)-2-(4-hydroxyphenyl)-2,3-dihydrobenzofuran-5-yl)vinyl)benzene-1,3-diol (*S,S*)-(+)-**5** **and 5-((*E*)-2-((2*R*,3*R*)-3-(3,5-dihydroxyphenyl)-2-(4-hydroxyphenyl)-2,3-dihydrobenzofuran-5-yl)vinyl)benzene-1,3-diol** (*R,R*)-(–)-**5**

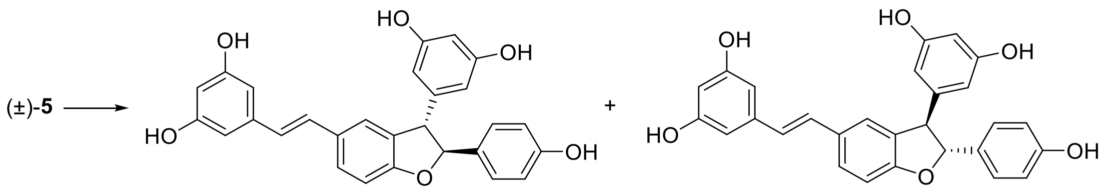

(2*S*,3*S*)-(+)-**5** (2*R*,3*R*)-(–)-**5**

The separation of the two enantiomers (*S*,*S*)-(+)-**5** and (*R*,*R*)-(–)-**5** was carried out by HPLC-UV using a chiral column (Kromasil 5-AmyCoat, 250 × 21.2 mm, λ = 280 nm) and an isocratic elution (hexane: *i*PrOH 70:30 + 0.1% TFA, rate flow 15 mL/min^{-1}). (2*S*,3*S*)-(+)-**5**, t_R= 6.3 min; $[\alpha]_D$ = +38 (*c* = 0.5, MeOH). (2*R*,3*R*)-(–)-**5**, t_R = 8.4 min; $[\alpha]_D$ = –38 (*c* = 0.5, MeOH).

5-((2*R*,3*R*)-6-hydroxy-2-(4-hydroxyphenyl)-4-((*E*)-4-hydroxystyryl)-2,3-dihydrobenzofuran-3-yl)benzene-1,3-diol and (2*R*,3*R*)-(+)-**6** **5-((2*S*,3*S*)-6-hydroxy-2-(4-hydroxyphenyl)-4-((*E*)-4-hydroxystyryl)-2,3-dihydrobenzofuran-3-yl)benzene-1,3-diol** (2*S*,3*S*)-(–)-**6**

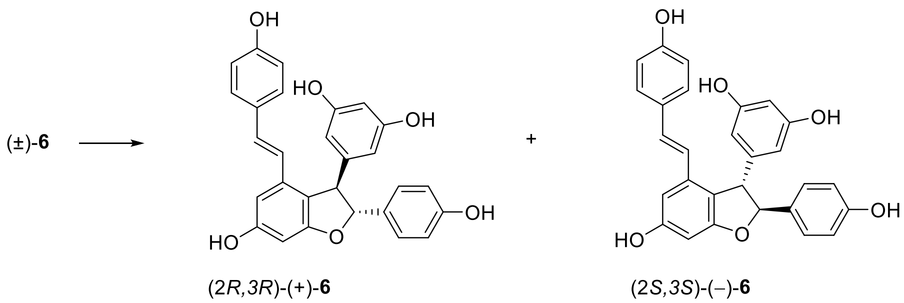

(2*R*,3*R*)-(+)-**6** (2*S*,3*S*)-(–)-**6**

The separation of the two enantiomers (2*R*,3*R*)-(+)-**6** and (2*S*,3*S*)-(–)-**6** was carried out by HPLC-UV using a chiral column (Kromasil-5 Ami Coat, 250×21.2 mm, λ = 280nm) and an isocratic elution (hexane: *i*PrOH 60:40 + 0.1% TFA, rate flow 15 mL/min^{-1}). (2*R*,3*R*)-(+)-**6**, t_R = 4 min; $[\alpha]_D$ = –29 (*c* = 0.3, MeOH). (2*S*,3*S*)-(–)-**6**, t_R= 6 min; $[\alpha]_D$ = +29 (*c* = 0.3, MeOH).

References (chemical synthesis)

22. Lee, H.S.; Lee, B. W.; Kim, M.R.; Jun, J.G. Syntheses of resveratrol and its hydroxylated derivatives as radical scavenger and tyrosinase inhibitor. Bull. Korean Chem. Soc. 2010, 31, 971–975. https://doi.org/10.5012/bkcs.2010.31.04.971.

23. Biasutto, L.; Marotta, E.; Mattarei, A.; Beltramello, S.; Calicet, P.; Salmaso, S.; Bernkop-Schnurch, A.; Garbisa, S.; Zoratti, M.; Paradisi, C. Absorption and metabolism of resveratrol carboxyesters. Cell. Physiol. Biochem. 2009, 24, 557–566. https://doi.org/10.1159/000257512.

24. Bernini, R.; Barontini, M.; Spatafora, C. New lipophilic piceatannol derivatives exhibiting antioxidant activity prepared by aromatic hydroxylation with 2-iodoxybenzoic acid (IBX). Molecules 2009, 14, 4669–4681. https://doi.org/10.3390/molecules14114669.

25. Han, S.Y.; Lee, H.S.; Choi D.H. Efficient total synthesis of piceatannol via (E)-selective Wittig-Horner reaction. Synth. Commun. 2009, 39, 1425–1432. https://doi.org/10.1080/00397910802528944.

26. Pezet, R.; Perret, C.; Jean-Denis, J.B.; Tabacchi, R.; Gindro, K.; Viret, O. δ-Viniferin, a resveratrol dehydrodimer: one of the major stilbenes synthesized by stressed grapevine leaves. J. Agric. Food Chem. 2003, 51, 5488–5492. https://doi.org/10.1021/jf030227o.

27. Vo, D.D.; Elofsson, M. Total synthesis of viniferifuran, resveratrol-piceatannol hybrid, anigopreissin A and analogues – Investigation of demethylation. Adv. Synth. Catal. 2016, 358, 4085–4092. https://doi.org/10.1002/adsc.201601089.

28. Snyder, S.A.; Breazzano, S.P.; Ross, A.G.; Lin, Y.; Zografos, A. L. Total synthesis of diverse carbogenic complexity within the resveratrol class from a common building block. J. Am. Chem. Soc. 2009, 131, 1753–1765. https://doi.org/10.1021/ja806183r.

29. Velu, S. S.; Buniyamin, I.; Ching, L. K. Regio- and stereoselective biomimetic synthesis of oligostilbenoid dimers from resveratrol analogues. Chem. - Eur. J. 2008, 14, 11376–11384. https://doi.org/10.1002/chem.200801575

Table S1. Docking score of top scoring complexes between pig pancreatic α-amylase and various resveratrol-derived compounds.

**Absolute configuration**	**Compound ID**	**docking score (kcal/mol)**
(*S*,*S*)-(+)	**5**	-8.144
(*S*,*S*)-(−)	**6**	-7.341
(*R*,*R*)-(−)	**5**	-6.908
(*R*,*R*)-(−)	**8**	-6.849
**-**	**4**	-6.427
**-**	**3**	-6.312
**-**	**1**	-6.041
(*S*,*S*)-(+)	**8**	-5.934
(*R*,*R*)-(+)	**6**	-5.851
